# The Multiple Functions of Insulin Put into Perspective: From Growth to Metabolism, and from Well-Being to Disease

**DOI:** 10.3390/ijms24010200

**Published:** 2022-12-22

**Authors:** Maria Elisabeth Street, Paolo Moghetti, Francesco Chiarelli

**Affiliations:** 1 Department of Medicine and Surgery, University of Parma, 43121 Parma, Italy; 2 Unit of Endocrinology, Diabetes & Metabolism, Department of Medicine, University of Verona, 37129 Verona, Italy; 3 Department of Pediatrics, University of Chieti, 66100 Chieti, Italy

Insulin has pleiotropic effects, and is of importance both as a key regulator of glucose metabolism and as a growth factor. Indeed, insulin is not only related to glucose tolerance, but has many other functions. Since the discovery of insulin, an enormous amount of knowledge has been acquired on the relationship between sensitivity/resistance and this hormone; thus, the pathways and networks involved in its signaling show specificities related to different organs, tissues and cell types. It is well known that changes in hormone sensitivity and circulating hormone concentrations are related to disease and growth. 

Several lines of evidence have proven the importance of insulin for growth [1]. Insulin is part of the insulin-like growth factor (IGF) system. Insulin-like growth factor (IGF)-I is the main peptide of this system, and its deficiency [2,3,4] is associated with pre- and postnatal growth deficiency, reduced brain development, and sensorineural deafness, in addition to a predisposition for high glucose levels in the later periods of life [5]. Gestational diabetes, characterized by high blood glucose and insulin levels alongside dyslipidemia, represents a strong example of how these metabolic changes modify the programming of most organs, including brain, liver, adipose tissue, muscle, cardiac muscle, spleen, and last, but not least, islet cell dysfunction, causing an overall increased risk of metabolic syndrome in adulthood [6]. 

Insulin resistance at birth may predispose a person to premature pubarche and polycystic ovarian syndrome [7]. Intrauterine growth restriction followed by rapid catch-up growth is also a condition characterized by insulin resistance that predisposes one to the development of metabolic syndrome [8], with features of insulin resistance being already present in the placenta of these individuals [9,10]. 

PCOS can also develop as a complication of obesity and insulin resistance. It is generally recognized that in most phases of life, insulin resistance in PCOS is associated with an increased risk of developing type 2 diabetes and, possibly, with an increased risk of cardiovascular disease [11]. 

Insulin receptor signaling shows significant cross-talks with the IGF-1 receptor type 1, and with FSH and LH signaling, acting as a co-gonadotropin in granulosa and theca cells. Furthermore, via known and unknown mechanisms in the presence of inflammation, such as changes in miRNAs and other epigenetic mechanisms, signaling can be easily affected. For example, CFTR malfunctioning, such as in cystic fibrosis, is associated with reduced FOXO1, leading to a molecular mechanism of insulin resistance [12] that goes beyond other known mechanisms mediated by inflammation and epigenetics [13]. 

Finally, the possible direct anti-inflammatory effects of insulin have never been fully elucidated, although some data have shown suggestions of an effect [14].

The present Special Issue sheds new light on the mechanisms of insulin resistance in relationship with specific endocrine conditions, such as hypogonadotropic hypogonadism in men where testosterone treatment alone seems not to be capable of restoring entirely normal energy production; this requires further and specific substrates, as the authors have shown using a metabolomic approach [15]. The growth hormone secretagogue receptor (GHS-R) has now been located on both α- and β-islet pancreatic cells. An elegant in vitro and in vivo study has proven the importance of this receptor for the regulation of glucose homeostasis and promises to be a potential candidate for the development of an antagonist that could work in type 2 diabetes [16]. Attention has been drawn to the mechanisms driven by myokines that lead to improved insulin sensitivity in skeletal muscle, and that play pivotal roles in type 2 diabetes [17]. The role of hyperinsulinemia and insulin resistance, driven mainly by nutrition, has been comprehensively analyzed by Joseph et al. [18] in relationship with disease, including cancer and aging, and it has been highlighted how future research and efforts should be made to guarantee normal insulin concentrations from birth. Physiologic insulin resensitization has been suggested as a treatment to revert the complications related to type 2 diabetes, and a scientific basis has been put forward for this [19]. In addition, the molecular mechanisms of action of metformin, as an insulin-sensitizing agent, particularly as related to the glucose transporter GLUT4, have been analyzed, shedding more light on the reasons for treatment and how it can work [20]. Finally, insulin resistance is long known to favor the onset of hypertension, particularly in patients with diabetes. The current guidelines and the pathophysiological pathways potentially implicated have been extensively revised by Tagi et al. [21]. 

In conclusion, insulin is a key hormone in human pathophysiology, playing roles that go far beyond glucose metabolism. These aspects currently deserve more attention within research and will hopefully lead to new treatments stemming from the research findings. 
